# Inflammation and Rho-Associated Protein Kinase-Induced Brain Changes in Vascular Dementia

**DOI:** 10.3390/biomedicines10020446

**Published:** 2022-02-14

**Authors:** Eun Chae Lee, Dong-Yong Hong, Dong-Hun Lee, Sang-Won Park, Ji Young Lee, Ji Hun Jeong, Eun-Young Kim, Hyung-Min Chung, Ki-Sung Hong, Se-Pill Park, Man Ryul Lee, Jae Sang Oh

**Affiliations:** 1 Department of Neurosurgery, College of Medicine, Cheonan Hospital, Soonchunhyang University, Cheonan 31151, Korea; lec9589@gmail.com (E.C.L.); dydehdghd@gmail.com (D.-Y.H.); madeby58@gmail.com (D.-H.L.); ppphilio3@gmail.com (S.-W.P.); applesori82@gmail.com (J.Y.L.); 2 Soonchunhyang Institute of Medi-Bio Science (SIMS), Soon Chun Hyang University, Cheonan 31151, Korea; jihun@sch.ac.kr; 3 Mireacellbio Co., Ltd., Seoul 04795, Korea; jlokey@miraecellbio.com (E.-Y.K.); hmchung@kku.ac.kr (H.-M.C.); kshong@miraecellbio.com (K.-S.H.); sppark@jejunu.ac.kr (S.-P.P.); 4 Department of Stem Cell Biology, School of Medicine, Konkuk University, Seoul 05029, Korea; 5 Faculty of Biotechnology, College of Applied Life Sciences, Jeju National University, Jeju 63243, Korea

**Keywords:** animal model, behavior test, biomarker, blood–brain barrier, cognitive dysfunction, inflammation, memory dysfunction, neuronal cell death, Rho-associated protein kinase, vascular dementia

## Abstract

Patients with vascular dementia, caused by cerebral ischemia, experience long-term cognitive impairment due to the lack of effective treatment. The mechanisms of and treatments for vascular dementia have been investigated in various animal models; however, the insufficient information on gene expression changes that define pathological conditions hampers progress. To investigate the underlying mechanism of and facilitate treatment development for vascular dementia, we established a mouse model of chronic cerebral hypoperfusion, including bilateral carotid artery stenosis, by using microcoils, and elucidated the molecular pathway underlying vascular dementia development. Rho-associated protein kinase (ROCK) 1/2, which regulates cellular structure, and inflammatory cytokines (IL-1 and IL-6) were upregulated in the vascular dementia model. However, expression of claudin-5, which maintains the blood–brain barrier, and MAP2 as a nerve cell-specific factor, was decreased in the hippocampal region of the vascular dementia model. Thus, we revealed that ROCK pathway activation loosens the tight junction of the blood–brain barrier and increases the influx of inflammatory cytokines into the hippocampal region, leading to neuronal death and causing cognitive and emotional dysfunction. Our vascular dementia model allows effective study of the vascular dementia mechanism. Moreover, the ROCK pathway may be a target for vascular dementia treatment development in the future.

## 1. Introduction

Vascular dementia is the second leading cause of dementia after Alzheimer’s disease (AD), and accounts for 30% of dementia cases in Asia. According to TOAST (trial of ORG 10,172 in acute stroke treatment), classifications of major stroke mechanisms, both in the US and Asia, exhibit a higher risk of ischemic stroke and vascular dementia [1,2,3].

Vascular dementia, unlike dementia caused by AD, is accompanied by extracranial stenosis or intracranial stenosis and it arises from hemodynamic insufficiency in the brain. While cerebral hypoxic damage is considered the main pathogenic factor in vascular dementia, the mechanism of vascular dementia has not been elucidated in prior research [4,5,6]. Vascular dementia is mediated by several mechanisms, including oxidative stress, altered cytokine and chemokine levels, and mitochondrial dysfunction [7]. The development of bilateral carotid artery stenosis is accompanied by gradual decreases in the cerebral blood flow. Once vascular insufficiency occurs, inflammatory factor levels increase and changes occur in the cytoskeleton [8]. The decrease in cerebral blood flow due to bilateral carotid artery ligation leads to excessive reactive oxygen species (ROS) [9] and inflammatory cytokine production in ischemic blood vessels, leading to antioxidant system inhibition [10], an inflammatory environment [11,12,13], and a low energy state, resulting in cell damage and mitochondrial dysfunction [14,15]. This was observed as cognitive decline due to nerve damage in the white matter [16], corpus callosum, and hippocampus [17] in both humans and animals. Inflammation is insufficient to explain the pathogenesis of vascular dementia. Additionally, the role of Rho-associated protein kinase (ROCK), an important marker of cytoskeletal changes, has been reported in some ischemia models, but has not been well-studied in vascular dementia.

In the present study, we aimed to elucidate the molecular mechanism underlying vascular dementia development by using an established animal model [18]. The bilateral carotid artery stenosis (BCAS) model is well-known in vascular dementia research and is considered sufficient for representing clinical vascular insufficiency in the brain [19]. In order to implement an accurate disease model, we generated a BCAS model and con-firmed, by behavioral analysis, that it represented early vascular dementia. Then, using this model, we investigated the pathogenesis and underlying mechanisms of vascular dementia based on the putative pathways of hypoxia-induced inflammatory blood–brain barrier (BBB) disruption, ROCK-induced cytoskeletal changes caused by vascular insufficiency, cell apoptosis, and structural changes in the cortex, corpus callosum, and hippo-campus.

## 2. Materials and Methods

### 2.1. Animals

All experimental procedures were performed at the Experimental Animal Center of the Soonchunhyang Institute of Medi-Bio Science (SIMS, Cheonan, South Korea). All animal experiments were performed in compliance with the Institutional Animal Care and Use Committee of Soon Chun Hyang University (IACUC No. SCH 20-0065) and the Guidelines for the Care and Use of Laboratory Animals specified by the National Research Council.

The experimental mice were housed in room with a 12-h light–dark cycle (7:00 a.m.–7:00 p.m.), with a temperature of 23 °C ± 1–2 °C and with a humidity of 50 ± 5%.

### 2.2. Experimental Design

**Figure 1:**
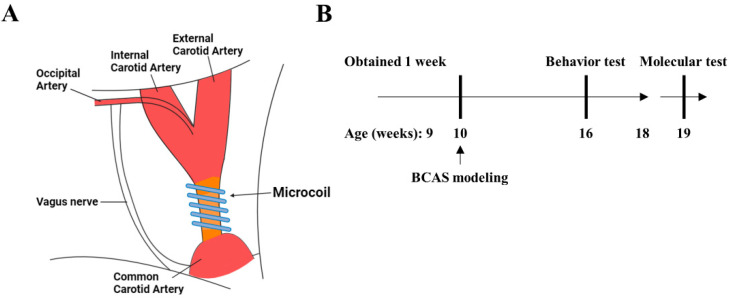
Modeling vascular dementia in a bilateral carotid artery stenosis (BCAS) mouse model. (**A**) The third rotation of the microcoil around the total carotid artery, after which the microcoil was tied. Blood vessel color change due to decreased blood flow due to microcoil ligation. (**B**) Schematic representation of the BCAS surgical strategy.

Female Balb/c nude mice (nine weeks old) were obtained from Orientbio (Seongnam, South Korea). After a one-week habituation period, we prepared a BCAS model in the 10-week-old mice. We designated the normal type (NT) group as the control group and designated the vascular dementia (VD) group as the experimental group. The experimental design was based on a previously reported study and was performed using microcoil implantation (SWPAO. 08 × 0.18 × 0.5 × 2.5, Samini Spring/Sawane, Shizuoka, Japan) (Figure 1A) [4]. Briefly, the mice were anesthetized with isoflurane (Hana Pharm, Seoul, South Korea). Mice were then fixed on the microscope (in the supine position), and a midline incision was made over the cervical region to expose their common carotid arteries (CCAs), which were then freed from their sheaths. We next carefully affixed microcoils to the carotid artery [20]. Finally, the wound was sutured with 6–0 silk. All processes were performed on a heating pad (25–26 °C). The mice were monitored for weight, body temperature, and paralysis twice weekly after surgery until stabilization occurred. Vascular dementia modeling was confirmed with behavioral analysis when mice were 16 weeks old. Molecular analyses were subsequently performed (Figure 1B).

### 2.3. Behavior Tests

All behavioral analyses were performed at the Experimental Animal Center of Soon-chunhyang Institute of Medi-Bio Science (SIMS, Cheonan, South Korea) and were con-ducted in a custom-made chamber (Scitech Korea, Seoul, South Korea) to reduce experi-mental deviation. White noise was present at a level of approximately 60 dB. Additionally, all experimental data (with the exception of the passive avoidance test) were analyzed using Smart v3.0 software (Panlab, Barcelona, Spain). Passive avoidance tests were con-ducted using a shut-avoidance program (Panlab, Barcelona, Spain). Statistically significant differences were confirmed using unpaired t-tests after conducting the experiments in triplicate.

#### 2.3.1. Y-Maze Test

A Y-maze (Gaon-Bio, Yongin, South Korea) was used to assess short-term memory and the locomotor activity index. The experimental time was eight min for each subject. Each mouse was initially placed in arm A (among zones A, B, and C, as well as the center of the maze box) and the correct alteration/total number of entries was recorded. The light intensity was set to 390 lx.

#### 2.3.2. Barnes-Maze Test

The Barnes-maze test (Gaon-Bio, Yongin, South Korea) evaluates learning, memory, and cognitive flexibility. The apparatus for this test was a circular black platform, 90 mm in diameter (18 holes). The experimental design was as follows. Block 1 included a training phase (three minutes to find and enter the escape route). Block 2 included a probe phase (removing the escape box and evaluating the time spent in the quadrant where the escape box had originally been located). Each mouse was placed in the middle of the apparatus. The time spent in each quadrant (i.e., the target zone, the target hole, and the error zone) were recorded. This experiment was repeated every day for four days. The light intensity was set to 390 lx.

#### 2.3.3. Passive Avoidance Test

The passive avoidance test (Harvard Apparatus, Holliston, MA, USA) is a test of long-term memory based on fear. The mouse was placed in a light compartment in the main box and was allowed a search time of 1 min. After 1 min, the door was opened, and the mouse entered a dark compartment. After the mouse entered the dark compartment for 2 s, the door was closed. The mouse was given an electric shock for 5 s. This experiment was repeated for four days. The light intensity in the box was set to 390 lx.

#### 2.3.4. Open Field Test

An open field test (Gaon-Bio, Yongin, South Korea) was used to assess motor activity and anxiety. Specifically, a 45 × 45 × 40-cm square open field was used for this test. The experimental time was 10 min for each subject. Each mouse was placed in the middle of the field. The center and peripheral zones were set to light intensities of 390 lx.

#### 2.3.5. Light and Dark Test

The light and dark test (Domestic) was used to assess locomotor activity and anxiety. The mice were placed in a 45 × 45 × 40 cm diameter square box with a black partition. The experimental time was 10 min for each subject. The large chamber was open and brightly illuminated (390 lx), while the small chamber was closed and dark. Each mouse was placed in the dark box. After 5 s, the door was opened. The experimental zones were maintained with the light and dark zones. The light intensity was set to 390 lx in the light zone.

### 2.4. Reverse-Transcription Quantitative Real-Time PCR

After being harvested, the frozen left hemisphere of each mouse (200 mg) was prepared and homogenized. To determine the mRNA expression levels of the target genes, total RNA was extracted from the mouse brain tissue using an easy-BLUE™ Total RNA Extraction Kit (iNtRON, Daejeon, Korea) according to the manufacturer’s protocol. The concentration and quality of the isolated RNA were determined using a NanoDrop spectrophotometer (NanoDrop Technologies, LLC; Wilmington, DE, USA). Next, cDNA was synthesized using 2 µg total RNA with an All-in-One 5 × First Strand cDNA Master Mix (CellScript, Madison, WI, USA), and quantitative real-time PCR (qRT-PCR) was performed using TOPreal™ qPCR 2X PreMIX (Enzynomics, Daejeon, Korea) according to the manufacturer’s protocol. Rock1, Rock2, Occludin, Claudin-5, Il-6, Il-1*β*, Mcp-1, Ccrr2, and VCAM-1 mRNA transcript levels were detected using the CFX Connect Real-Time PCR Detection System (Bio-Rad, Hercules, CA, USA).

### 2.5. Immunohistochemical Staining

The paraffin-embedded sections were dewaxed with xylene and dehydrated with a graded alcohol series. Subsequently, sections were incubated in 3% (*w*/*v*) H_2_O_2_ for 2 min and washed with PBS three times for 5 min each. Next, antigens were retrieved with 10 mM sodium citrate buffer. The sections were treated with peroxidase for 10 min in blocking solution to block endogenous peroxidase, and then in 5% goat serum for 10 min to block non-specific antibody binding. Overnight incubation with rabbit anti-alpha smooth muscle actin (α-SMA) polyclonal antibody (1:300; abcam, Boston, MA, USA) or rabbit anti-microtubule-associated protein 2 (MAP2) polyclonal antibody (1:500; GeneTex, Irvine, CA, USA) was performed in humidified boxes at 4 °C. Phosphate-buffered saline (PBS) was used as a negative control. Staining was then developed with a 3,3′-diaminobenzidine (DAB) solution for 3 min. Tissues were rinsed in PBS three times, for 5 min each time, between each step, and then stained with hematoxylin. Sections were subsequently mounted, dehydrated, coverslipped, and examined under a Motic Easyscanone (Houston, TX, USA). Immunohistochemistry was analyzed with an Motic Digital Slide assistant system (Motic China Group Co., Ltd., Xiamen, China).

### 2.6. Luxol Fast Blue Staining

Luxol fast blue (LFB) staining was performed on brain sections to visualize myelin tracts in the corpus callosum. After rehydration in distilled water, the sections were stained with LFB solution for 2 h at 60 °C followed by differentiation in 0.05% lithium carbonate solution and 70% ethanol for 45 s to 2 min until the gray matter was colorless while the white matter stained blue and was sharply defined. The sections were then washed in distilled water, dehydrated, cleared, and coverslipped.

### 2.7. Statistical Analysis

Statistical analyses were performed using GraphPad Prism 8 software (GraphPad, Inc., San Diego, CA, USA). Data are presented as means ± standard deviations (SD). For comparisons involving more than two groups, all analyses were performed at least in triplicate and statistical differences were analyzed via unpaired *t*-tests. Two-sided *p*-values of <0.05 were considered statistically significant.

## 3. Results

### 3.1. Behavior Testing Results

**Figure 2:**
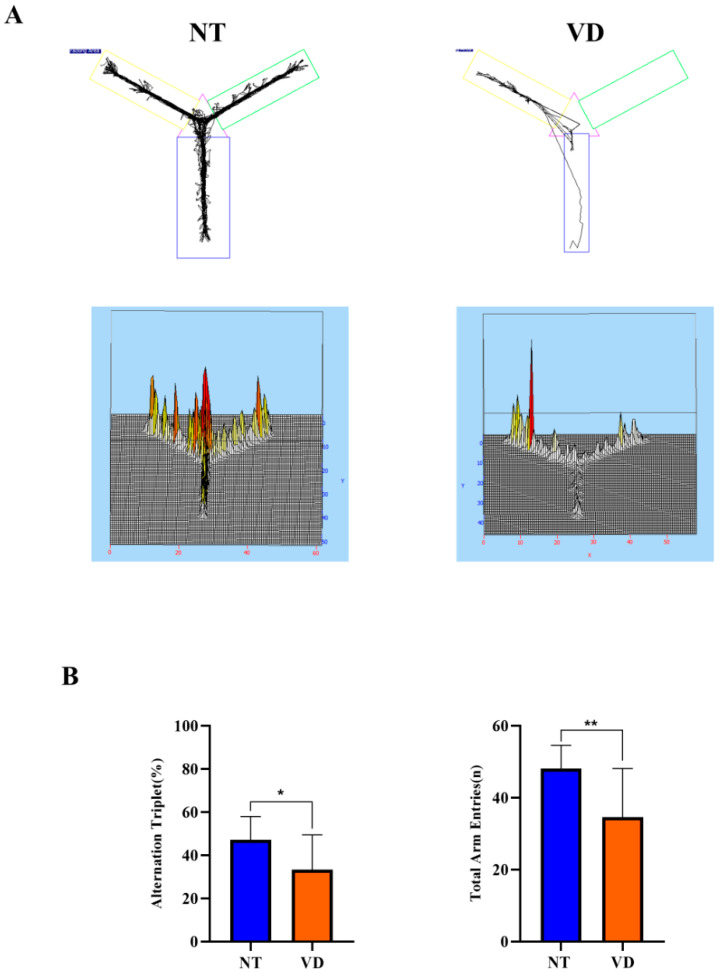
Y-maze test (normal type [NT]: control group and blue color graph, vascular dementia [VD]: BCAS [bilateral carotid artery stenosis] model group and madarin color graph). (**A**) Recorded experimental data. The start zone is the blue box. (**B**) Differences in the alternation triplet percentage between groups (left). Differences in the total number of arm entries between groups (right). Data are expressed as means ± standard deviations (SD). (* *p* < 0.05; ** *p* < 0.01).

In the NT group, short-term spatial memory measurements were obtained using the Y-maze test [16,21,22]. However, BCAS mice showed repetitive behavior with memory impairment, entering the same arm repeatedly (Figure 2A). We found that the percentage was lower in the VD group than in the NT group (NT 48.2% vs. VD 34.6%; *p* < 0.05, Figure 2B, Table 1). Moreover, the total number of arm entries during the test was considerably greater in the NT than in the VD group (NT 48.1% vs. VD 34.6%, *p* < 0.04, Figure 2B, Table 1). The higher percentage of alternation triplets in the BCAS model demonstrated a tendency for these mice to explore new environments; thus, our study conclusively confirmed a protocol for building and evaluating a BCAS mouse model [21].

**Figure 3:**
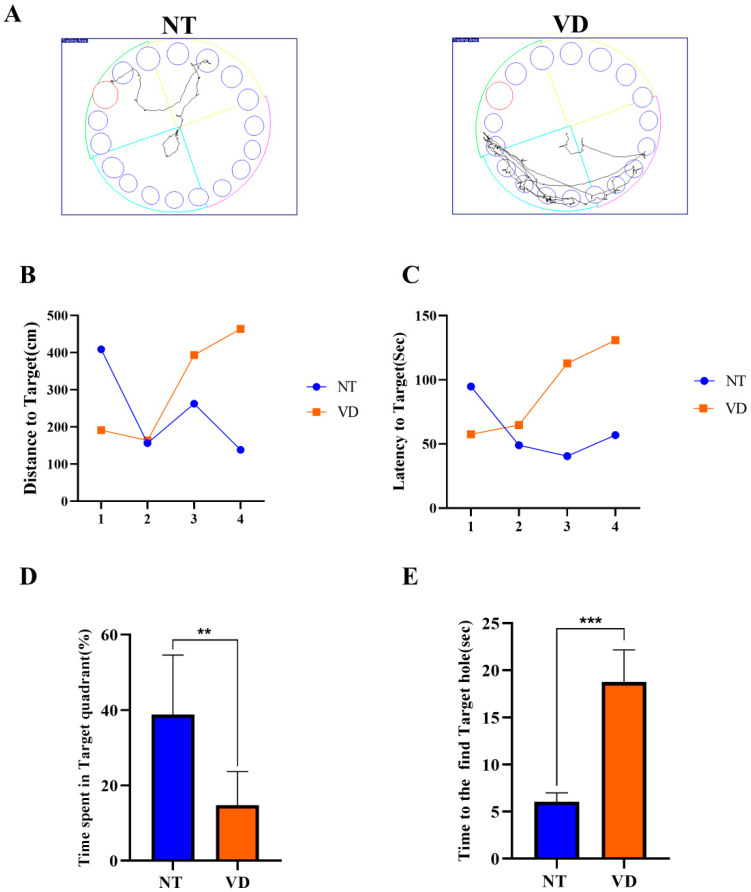
Barnes-maze test (NT [normal type]: control group, VD [vascular dementia]: BCAS [bilateral carotid artery stenosis] model group). (**A**) Recorded experimental data. The target zone was designated via a red circle and the error zone was designated via a blue circle (left: NT, right: VD). (**B**) Differences between groups for the distance to the target (by day). (**C**) Differences between groups for latency to the target (by day). (**D**) Differences between groups in the percentage of time spent in the time quadrant ** *p* < 0.01. (**E**) Differences between groups in the time spent to find the target hole in the time quadrant *** *p* < 0.001. Data are expressed as means ± standard deviations (SD).

The Barnes-maze test was used to assess spatial learning and memory impairment (Figure 3A) [23,24]. Repeated experiments were conducted over the course of four days to determine if the location of the food was remembered through several instances of repeated learning. In the NT group, the distance and time to find the target was decreased on the last day as compared to the first day, whereas the time to find the target was increased in the VD group (Figure 3B,C, Table 2). During exploratory trials, the VD group animals spent a statistically significant less amount of time in the target quadrant (where the escape box had previously been located; NT 38.84% vs. VD 14.7%, *p* < 0.01, Figure 3D, Table 2). Based on comparison of the time required to find the target hole (NT, 6 s vs. VD, 18.7 s, *p* < 0.01, Figure 3E, Table 2), we concluded that VD group mice found it difficult to locate the target hole, despite repeated training.

**Figure 4:**
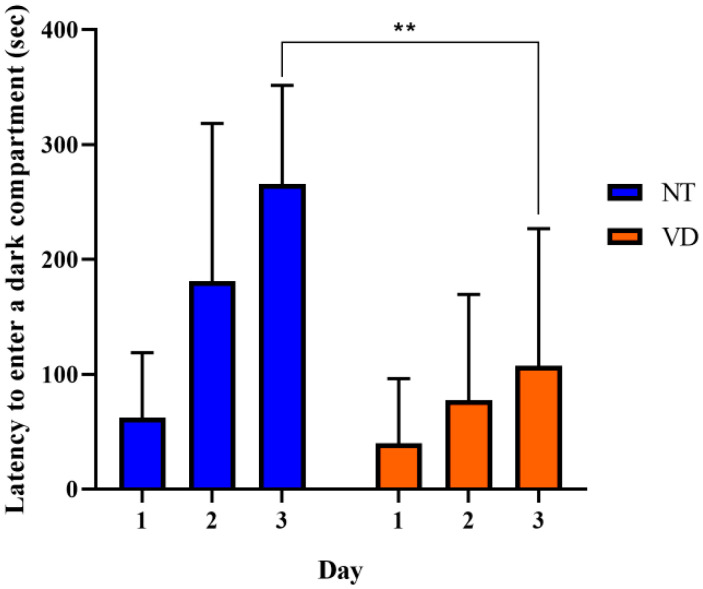
Passive avoidance test. Negative memory was evaluated based on the mean latency time (s) to enter the electric shock box. (NT [normal type]: control group, VD [vascular dementia]: BCAS [bilateral carotid artery stenosis] model group). Data are expressed as means ± standard deviations (SD), ** *p* < 0.01.

The passive avoidance test was used to evaluate negative learning and long-term memory by repeatedly evoking entrapment. When the mice remembered the stress induced by the electrical shock in the text box, the time spent in the test box differed between the NT and VD groups (Figure 4, Table 3) [16,22,25]. In the VD group, memory was not established, despite repetitive learning, whereas in the NT group, memory was maintained over time. On day three, we found a statistically significant difference between the VD and NT groups (VD 265.7 s vs. NT 107.4 s). Thus, BCAS modeling could induce mice to step into the electric shock box more frequently, confirming that fear avoidance and hippocampus-dependent contextual memory were degraded in these model mice.

**Figure 5:**
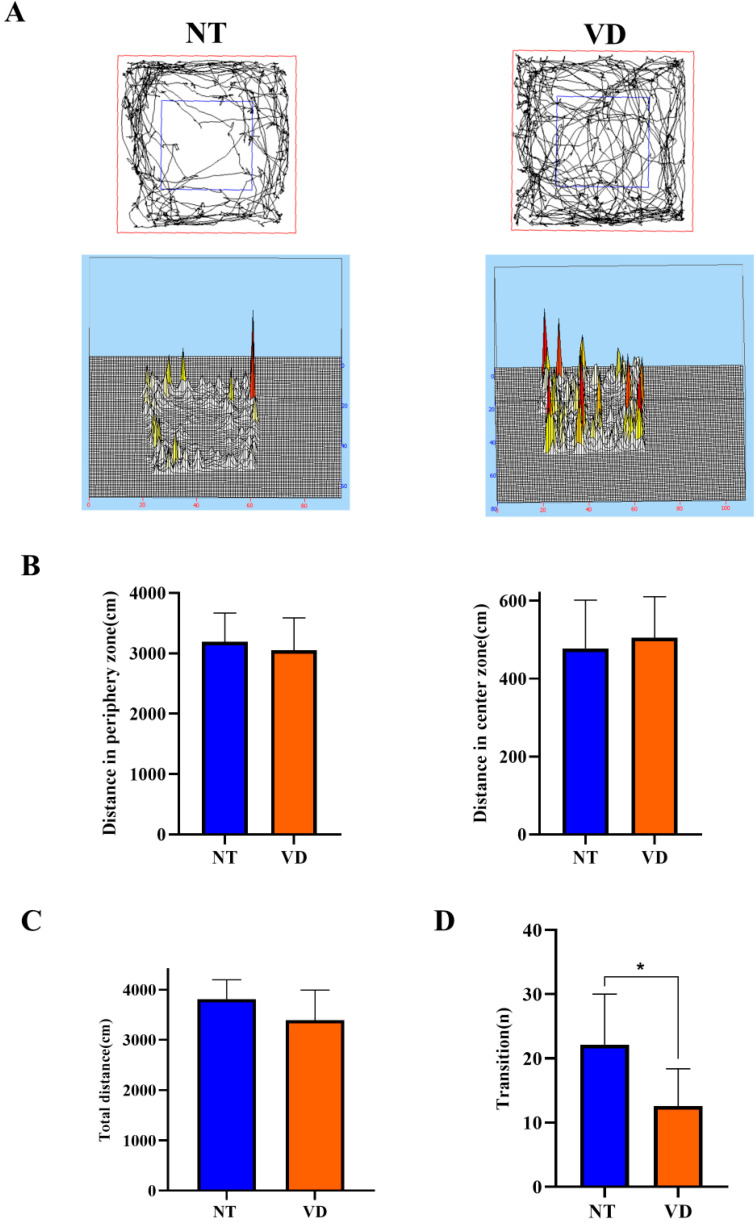
A depiction of the open field test and the light and dark test (NT [normal type]: control group, VD [vascular dementia]: BCAS [bilateral carotid artery stenosis] model group). (**A**) Smart 3.0 analysis program data (left: NT, right: VD). (**B**) Differences in the distance moved between the peripheral zone (NT, 3192.1 cm; VD, 3050.2 cm) and the central zone (NT, 483 cm; VD, 521.8 cm) (**C**) Differences between groups in the total distance moved in the open field test. (**D**) Differences between groups in the number of transitions in the light/dark transition test, * *p* < 0.05. Data are expressed as means ± standard deviations (SD).

In the open field test (an anxiety test for mice), the NT group spent more time in the peripheral area than in the central area (Figure 5A) [26,27]. In the peripheral zone, the VD group moved a shorter distance than did the NT group (Figure 5B, left, Table 4). In the central area, the NT group showed a longer movement distance than did the VD group (Figure 5B, right, Table 4). Total movement distance was only slightly different between the VD and NT groups (Figure 5C, Table 4). The light/dark transition test confirmed that mice in the VD group were less anxious than the NT mice, given that mice demonstrate more anxiety in bright light (Figure 5D, Table 4) [28,29] (NT 22.1 vs. VD 12.5, *p* < 0.0113).

These behavioral analyses confirmed that the BCAS animal model demonstrated clinical characteristics of human vascular dementia.

### 3.2. Increases in ROCK Expression in the Brains of VD Model Mice

**Figure 6:**
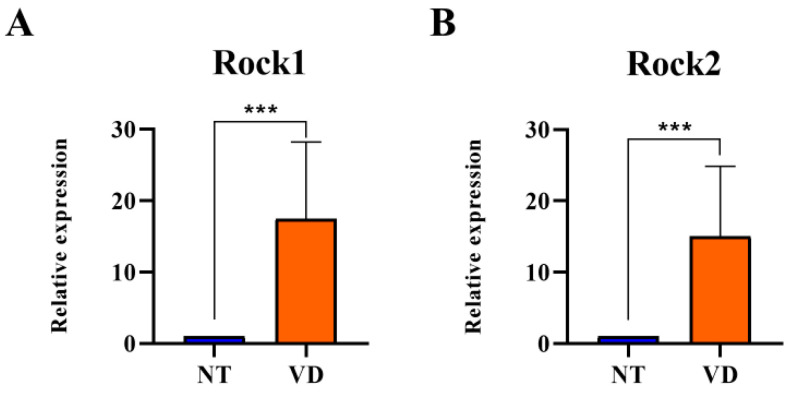
Rock mRNA expression in the brain of bilateral carotid artery stenosis (BCAS) mice. (NT [normal type]: control group, VD [vascular dementia]: BCAS [bilateral carotid artery stenosis] model group). (**A**,**B**) mRNA expression of ROCK1/2 was determined by quantitative real-time polymerase chain reaction in the two groups (NT, VD) after BCAS. *** *p* < 0.001. The results are expressed as mean values and the error bars represent standard deviations.

Next, to identify the specific mechanism associated with induced vascular dementia, we isolated the brains of the VD model mice and used RT-qPCR to determine mRNA transcription levels of ROCK in the NT and VD groups to identify the mechanisms causing ischemic brain injury in VD mice. ROCK regulates actin cytoskeletal reorganization and interaction with tight junction (TJ) proteins in endothelial cells. ROCK mRNA levels were higher in the VD group than in the NT group (Rock1: 1.00 [NT] vs. 17.48 [VD], *p* = 0.0003; Figure 6A, Table 5; Rock2: 1.00 [NT] vs. 15.01 [VD], *p* = 0.0006, Figure 6B, Table 5). Therefore, BCAS statistically significantly increased Rock expression, suggesting that TJ protein redistribution occurred in BCAS-induced brain injury.

### 3.3. Reduction in TJ Protein Expression in the Brain of VD Model Mice

**Figure 7:**
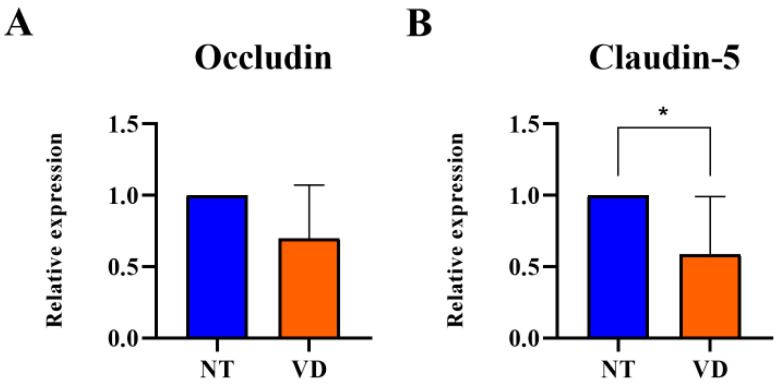
Quantitative real-time polymerase chain reaction analysis revealed that bilateral carotid artery stenosis (BCAS) induced decreased mRNA levels of Occludin and Claudin-5 in the VD group. (NT [normal type]: control group, VD [vascular dementia]: BCAS [bilateral carotid artery stenosis] model group). (**A**,**B**) Relative mRNA expressioin levels of Occludin and Claudin-5. * *p* < 0.05. The results are represented as mean values and the error bars represent  standard deviations.

Increased expression of ROCK in VD mice indicated a decrease in TJ-related proteins. In brain vascular endothelial cells, TJs form barriers that limit cell permeability. After BCAS, the mRNA expression levels of Occludin and Claudin-5, which encode proteins in-volved in TJs, were statistically significantly reduced in the BCAS group as compared to the NT group (Occludin: 1.00 [NT] vs. 0.69 [VD], *p* = 0.0745, Figure 7A, Table 6; Claudin-5: 1.00 [NT] vs. 0.58 [VD], *p* = 0.0300, Figure 7B, Table 6). BCAS disrupted the BBB through decreased endothelial adhesion junction protein expression and increased ROCK expression. The decrease in the expression of Occludin and Claudin-5 indicates the collapse of the BBB structure, which is likely to increase inflammatory reactions.

### 3.4. Increased Expression of Adhesion Molecules and Pro-Inflammatory Cytokines in VD Mice

**Figure 8:**
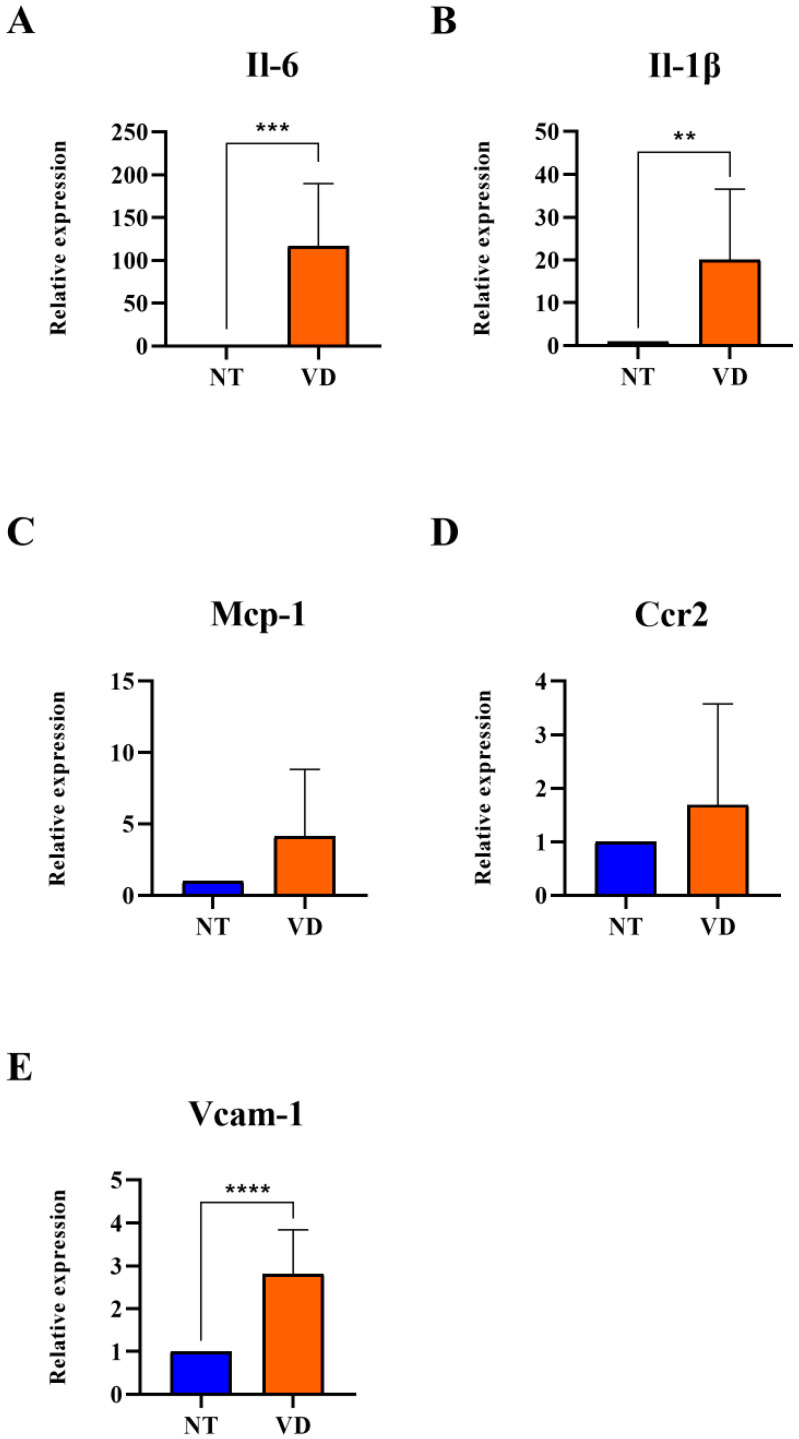
Upregulated expression of adhesion molecules and pro-inflammatory cytokines after BCAS (NT [normal type]: control group, VD [vascular dementia]: BCAS [bilateral carotid artery stenosis] model group) (**A**–**E**) Relative mRNA expression levels of IL-6, IL-1*β*, Mcp-1, Ccr2, and VCAM-1. ** *p* < 0.01, *** *p* < 0.001, and **** *p* < 0.001. The results are presented as mean values and the error bars standard deviations.

Vascular cell attachment molecules-1 (VCAM-1) control the occurrence and amplification of tissue inflammation during ischemic brain damage. Many inflammatory factors are elevated in brain diseases when the BBB is damaged. Inflammatory factors bound to white blood cell ligands by cell adhesion molecules migrate to the injured brain tissue [30,31,32,33]. In this study, the change in mRNA expression in the pro-inflammatory milieu of the BCAS model was confirmed by RT-qPCR. VCAM-1, Il-6, Il-1β, Mcp-1, and Ccr2 were statistically significantly higher in the VD group than in the NT group (VCAM-1: 1.00 [NT] vs. 2.82 [VD], *p* < 0.0001, Figure 8E, Table 7; Il-6: 1.00 [NT] vs. 116.7 [VD], *p* < 0.0007, Figure 8A, Table 7; Il-1β: 1.00 [NT] vs. 20.08 [VD], *p* < 0.0058, Figure 8B, Table 7; Mcp-1: 1.00 [NT] vs. 4.12 [VD], *p* < 0.0635, Figure 8C, Table 7; Ccr2: 1.00 [NT] vs. 1.69 [VD], *p* = 0.3846, Figure 8D, Table 7). These results suggested that increased inflammatory reactions occur through the expression of high levels of adhesion molecules and pro-inflammatory cytokines associated with pathological conditions in the VD group.

### 3.5. Vasoconstriction and Apoptosis in the VD Group

**Figure 9:**
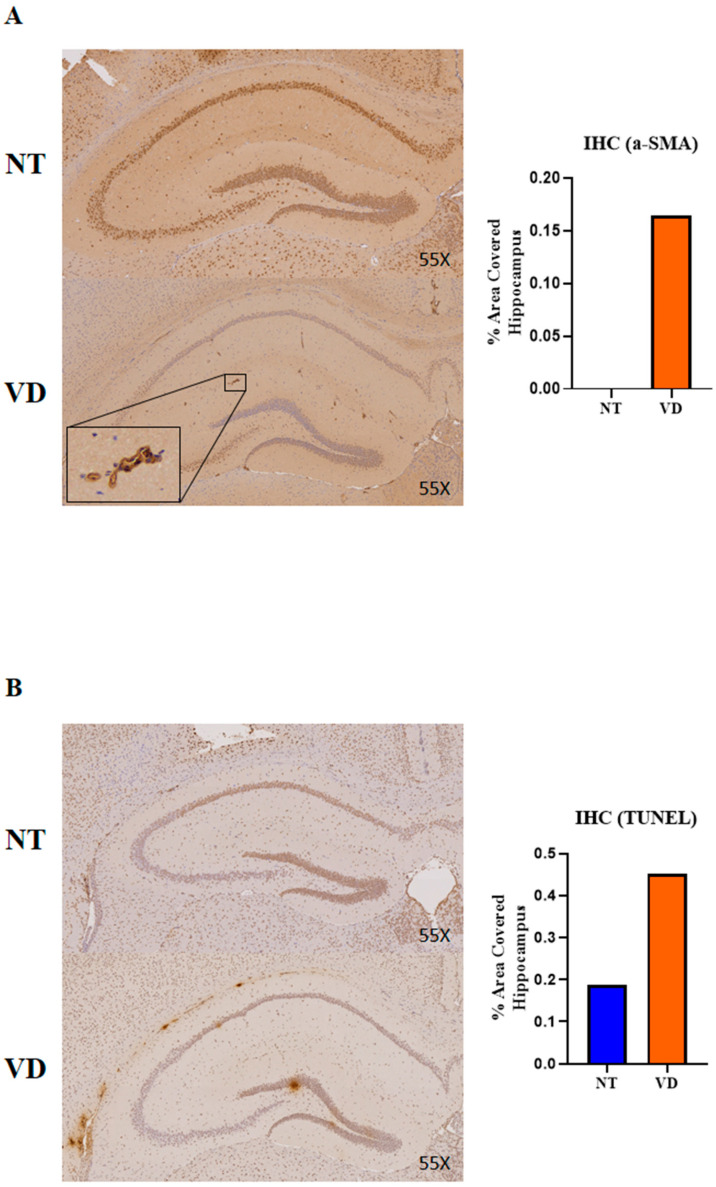
Immunoreactivity of alpha smooth muscle actin (α-SMA) and results of a terminal deoxynucleotidyl transferase dUTP nick end labeling (TUNEL) assay in the hippocampus in the NT and VD groups (NT [normal type]: control group, VD [vascular dementia]: BCAS [bilateral carotid artery stenosis] model group). (**A**,**B**) Left: representative photomicrographs of α-SMA in TUNEL immunoreactive cells. Right: quantification of α-SMA through TUNEL immunoreactivity.

α-SMA induces chronic angiopathy in obstructive vascular diseases, including is-chemic stroke. The terminal deoxynucleotidyl transferase dUTP nick end labeling (TUNEL) assay detects DNA fragments generated during cell apoptosis. In the VD mouse brains, α-SMA and TUNEL were confirmed by immunohistochemistry (IHC). The VD group showed statistically significantly higher α-SMA (Figure 9A, Table 8) and TUNEL (Figure 9B, Table 8) staining than the NT group. These results suggest that BCAS can induce brain damage by increasing α-SMA expression in the hippocampus, as well as by inducing an increase in vasoconstriction and apoptosis.

### 3.6. Changes in Brain Structure in the VD Group

**Figure 10:**
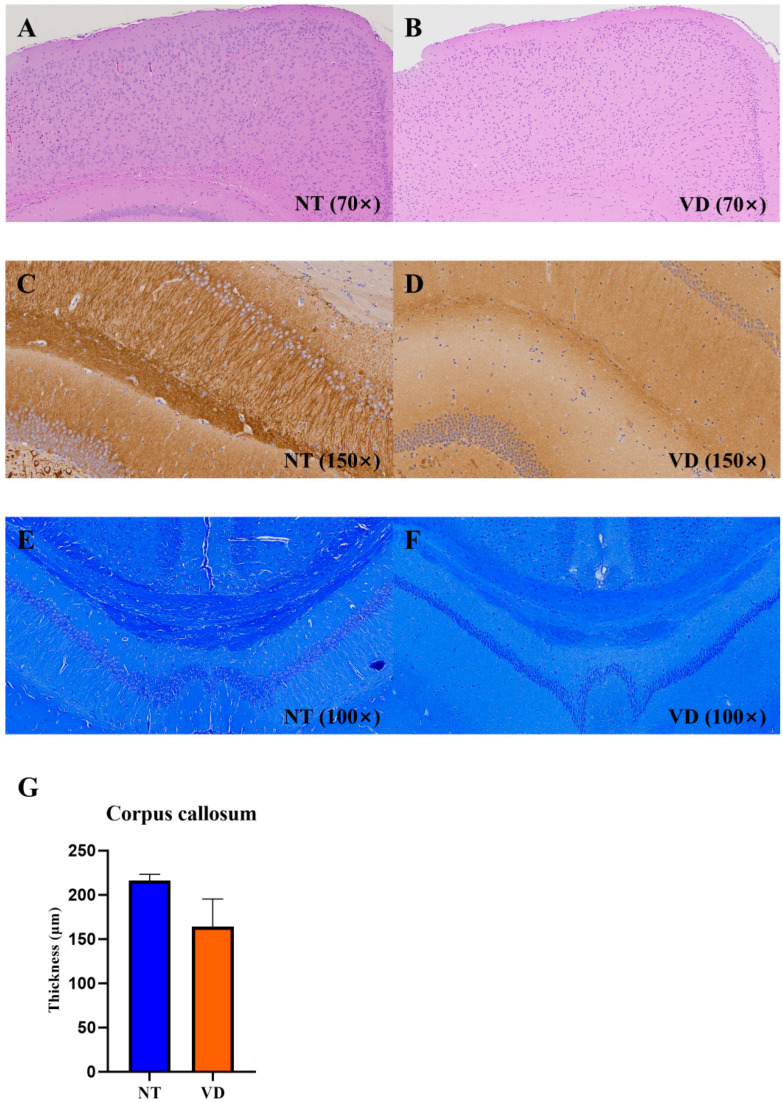
Hematoxylin and eosin (H&E), immunohistochemistry (IHC; MAP2), and Luxol fast blue staining in the brains of the VD group. NT [normal type]: control group, VD [vascular dementia]: BCAS [bilateral carotid artery stenosis] model group). (**A**,**B**) H&E staining in the NT and VD groups. (**C**,**D**) MAP2 IHC in the NT and VD groups (hippocampal cornu ammonis [CA1] region). (**E**,**F**) Myelin staining. Coronal sections of paraffin-embedded brains from the NT and VD groups were stained with Luxol fast blue. All images are of the cerebral cortex (left) and the hippocampus (right). (**G**) Thickness of the corpus callosum in NT and in VD mouse tissue, obtained using Image J software.

The cerebral cortex is a collection of neurons located on the surface of the cerebrum. The corpus callosum consists of thick bundles of nerve fibers that connect to the cerebral hemispheres, allowing interhemispheric conduction of signals [34,35]. In the VD group, irregularities in cortical arrangement (Figure 10A,B) and decreased MAP2-positive neurons (Figure 10C,D) in the cornu ammonis (CA1) region of the hippocampus were confirmed. LFB staining appeared uniformly throughout the corpus callosum in the NT group, but was not uniformly found in the VD group (Figure 10E,F, Table 9) [36]. The corpus callosum thickness was reduced in the VD group as compared to the NT group [37]. These results suggest increased cortical, hippocampal, and corpus callosum damage in the VD group, due to decreased cerebral blood flow.

## 4. Discussion

**Figure 11:**
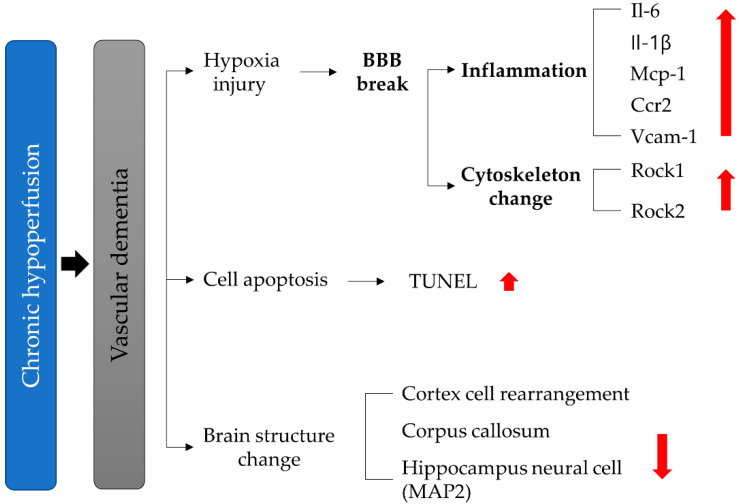
Schematic representation of vascular dementia.

Chronic hypoperfusion has two effects. First, a hypoxic injury phenomenon is induced, which can cause BBB collapse, which results in inflammation and cytokine changes. It is already established that an ischemic dementia model can elucidate the mechanisms of neuronal death and dysfunction after ischemia. In the present study, we established a vascular insufficiency model (a BCAS model) using a microcoil [38,39,40]. The low ischemia was induced by occlusion of the common carotid and vertebral arteries, resulting in inflammation and BBB disruption in the absence of motor weakness. Results from our study identified behavioral deficits in the VD model. The results of the Y-maze and Barnes-maze confirmed that spatial learning, short-term memory, spatial cognitive ability, and cognitive ability were adversely affected by BCAS in the VD group [41,42,43,44]. As a confirmation of early vascular dementia, we found that cell changes in CA1 levels in the hippocampus occurred at 6 weeks post-BCAS. Changes in the nucleus and cell rearrangement in the brain cortex, corpus callosum, and white matter tract were similarly confirmed [16,45]. We then investigated the pathogenesis and mechanisms for vascular dementia according to the putative pathways of hypoxia-induced inflammatory BBB disruption: ROCK-induced cytoskeletal changes due to vascular insufficiency, apoptosis, and structural changes in the cortex, corpus callosum, and hippocampus (Figure 11), and showed gene expression changes that reflected these events. BBB disruption is a pathological hallmark of ischemic brain injury [46]. However, the mechanism underlying this process remains unclear. In our study, we found that increased Rock and inflammatory factors accompanied BBB disruption (Figure 6, Figure 7 and Figure 8). Our findings suggest that ROCK, adhesion molecules, and pro-inflammatory cytokines are important physiological and pathological modulators.

Ischemic brain injury is accompanied by increased inflammation as well as increased BBB permeability [47]. In the ischemic brain, inflammatory mediators stimulate cerebral endothelial cells to induce inflammatory responses [48,49]. Expression of the VCAM-1 adhesion molecule has been comprehensively studied as an indicator of inflammation in models of cerebral ischemia [50]. Our results indicated an increase in VCAM-1 levels after the BCAS group. Additionally, the major pro-inflammatory cytokines evaluated in this study were Il1, Il6, and Mcp1 [47,51]. These cytokines are initiators of the inflammatory response and promote the expression of adhesion molecules [52], suggesting that pro-inflammatory cytokines upregulated adhesion molecules and aggravated brain injury in the VD mice in our study [53].

The BBB is required for the structural stabilization of TJ proteins as structural com-ponents that maintain cerebrovascular integrity and BBB function [54,55]. Activated ROCK and pro-inflammatory cytokines correspond to vascular endothelial damage [51,56]. We found an inflammatory response that was upregulated by higher vascular permeability in the ischemic-injured brains of the VD group (Figure 8A–E).

The cerebral cortex is the largest site of neural integration in the central nervous sys-tem, and plays an important role in memory, language, and consciousness. When the blood supply to the brain is reduced due to narrowing of the brain arteries, the brain tissue suffers ischemic damage. [41,44,57]. In the BCAS group, we confirmed that the cortical cell arrangement was altered and demonstrated irregular cell shapes. These results suggested damage to the cortex, which is responsible for cognitive function (Figure 10A,B).

MAP2, a cytoskeletal microtubule-associated protein distributed in the hippocampal region which is responsible for learning and memory processes, plays an important regulatory role in maintaining neuronal plasticity and differentiated neuron morphology. The statistically significant decrease in MAP2-dependent neuronal plasticity and structural integrity in the hippocampal CA1 region of the VD group seen in this study suggests a detrimental effect on the ability to learn new facts and store memory (Figure 10C,D).

The corpus callosum is a myelinated structure that acts as a bridge between the brain hemispheres and is responsible for signal transmission. Damage to the corpus callosum disrupts contact between the two hemispheres, leading to various impairments (e.g., language and cognitive functions). Myelin staining of the corpus callosum was decreased in the VD group and was not uniform, as compared with the NT group. The corpus callosum thickness was also decreased in the VD group, suggesting a decrease in the corpus callosum myelin and cognitive decline in the VD model group [58] (Figure 10E,F).

Many previous studies of cognitive disorders used a model of acute ischemic conditions, such as middle cerebral artery occlusion. This model focused on acute neuronal injury and death after an abrupt decrease in cerebral blood flow or complete cessation of cerebral perfusion. Occlusion of the cerebral arteries quickly induced the neuronal cell loss in the hippocampus, directly resulting in cognitive impairment. However, this model cannot represent patients with other causes of VD, such as large artery atherosclerosis, an etiology more common than acute cerebral ischemia. Our study used one of the models of chronic progressive hypoperfusion of the brain. Many previous studies investigating novel treatments for VD failed because the pathogenesis of VD was unclear and appropriate animal models had not yet been developed. Our study reveals the parallel effect of the ROCK pathway and inflammation on VD development, confirmed through behavioral tests. Our results demonstrated the potential of ROCK, pro-inflammatory cytokines, and the BBB status to be early markers of vascular dementia. Moreover, the potential improvement in vascular pathology through ROCK inhibitors or stem cell treatment warrants further investigation. Thus, our findings indicate directions for further research into early- and later-stage vascular dementia research and may ultimately inform medical guidelines.

## 5. Conclusions

Vascular dementia is chronic, presenting with persistent hypoperfusion injury [59]. Chronic hypoperfusion injury causes a long-term decrease in blood flow, resulting in cognitive impairment rather than in severe neurological impairment in the brain. We found that the BCAS VD mouse model established in this study reflects clinical characteristics of the vascular dementia disease course. More specifically, we confirmed the underlying molecular mechanism of vascular dementia to be as follows: In vascular dementia, an in-crease in ROCK levels leads to a decrease in TJ proteins (Occludin, Claudin-5) that form part of the BBB, which regulates vascular permeability, by inducing restructuring of the cytoskeleton. This induces neuroinflammation through the BBB, with increased vascular permeability, and an inflammatory environment in which pro-inflammatory cytokines and chemokine levels (e.g., VCAM-1, IL-6, IL-1*β*, MCP-1, and CCR2) are increased. This causes damage to the (1) hippocampus, which is responsible for learning and memory; (2) the cerebral cortex, which is responsible for long-term memory; and (3) the corpus callosum, which functions in interhemispheric integration, which explains the decrease in cognitive function and memory in the BCAS group. Our study showed pathological changes leading to early vascular dementia and causing it to become chronic. This can mimic the pathological phenomenon seen in patients with early vascular dementia.

## Figures and Tables

**Table 1:** Y-maze tests and animal assignments.

	Group	No. of Mice	Mean Value
Y-maze (Alternation triplet)	NT	9	47.22
	VD	9	33.29 *
Y-maze (Total arm entry)	NT	9	48.13
	VD	9	34.61 **

Values are means ± standard deviations. * *p* < 0.05 and ** *p* < 0.01 represent the tendency and significant difference, respectively.

**Table 2:** Barnes-maze tests and animal assignments.

	Group	No. of Mice	Mean Value
Barnes-maze (Time spent in target quadrant)	NT	9	38.84
	VD	8	14.74 **
Barnes-maze (Time to the find target hole)	NT	17	6.060
	VD	9	18.73 ***

Values are means ± standard deviations. ** *p* < 0.01 and *** *p* < 0.001 represent the tendency and significant difference, respectively.

**Table 3:** Passive avoidance test and animal assignments.

	Group	No. of Mice	Mean Value
Passive avoidance	NT	9	38.84
	VD	8	14.74 **

Values are means ± standard deviations. ** *p* < 0.01 represents the tendency and significant difference.

**Table 4:** Open field, light and dark test, and animal assignments.

	Group	No. of Mice	Mean Value
Open field (Distance in periphery zone)	NT	9	3192
	VD	8	3050
Open field (Distance in center zone)	NT	11	477.6
	VD	10	504.8
Open field (Total distance)	NT	12	NS
	VD	9	
Light and dark (Transition)	NT	13	22.15
	VD	7	12.57 *

Values are means ± standard deviations * *p* < 0.05 represents the tendency and significant difference.

**Table 5:** List of the experimental groups (Rock1/2).

	Group	No. of Mice	Mean Value
Rock1	NT	9	1
	VD	9	17.48 ***
Rock2	NT	9	1
	VD	9	15.01 ***

Values are means ± standard deviations. *** *p* < 0.001 represents the tendency and significant difference.

**Table 6:** Occludin and Claudin-5.

	Group	No. of Mice	Mean Value
Occludin	NT	9	1
	VD	9	0.69
Claudin-5	NT	9	1
	VD	9	0.58 *

Values are means ± standard deviations. * *p* < 0.05 represents the tendency and significant difference.

**Table 7:** Pro-inflammatory cytokines.

	Group	No. of Mice	Mean Value
Il-6	NT	9	1
	VD	9	116.7 ***
Il-1β	NT	9	1
	VD	9	20.08 **
Mcp-1	NT	9	1
	VD	9	4.12
Ccr2	NT	9	1
	VD	9	1.69
Vcam-1	NT	9	1
	VD	9	2.82 ****

Values are means ± standard deviations. ** *p* < 0.01, *** *p* < 0.001, and **** *p* < 0.0001 represent the tendency and significant difference, respectively.

**Table 8:** α-SMA and TUNEL.

	Group	No. of Mice	Mean Value
α-SMA	NT	3	0
	VD	1	0.165
TUNEL	NT	1	0.187
	VD	1	0.451

Values are means ± standard deviations.

**Table 9:** Corpus callosum.

	Group	No. of Mice	Mean Value
Corpus callosum	NT	3	216.2
	VD	2	163.9

Values are means ± standard deviations.

## Data Availability

No new data were created or analyzed in this study. Data sharing is not applicable to this article.
